# Progressive spinal cord compression technique in experimental rabbit animal model for cervical spondylotic myelopathy

**DOI:** 10.1016/j.amsu.2021.102603

**Published:** 2021-07-28

**Authors:** Sabri Ibrahim, Wibi Riawan

**Affiliations:** a Department of Neurosurgery, Medical Faculty Universitas Sumatera Utara, Medan, Indonesia; b Department of Biochemistry and Biology Molecular, Medical Faculty Universitas Brawijaya, Malang, Indonesia

**Keywords:** Cervical spondylotic myelopathy, Spine, GFAP, S100-β, Neurofilaments

## Abstract

**Introduction:**

Cervical spondylotic myelopathy (CSM) presently estimated at 54% population, commonly cause of myelopathy due to chronic compression of the spinal cord in older people. Physiological injuries caused by static and dynamic forces including compressed, pinched, and pulled out inducing secondary injuries at the molecular level.

**Methods:**

We examined the rabbit model approach with the clinical case of spondylotic myelopathy, in which the disk and facet maintained the cervical spine mobility, and compression was given 0.5 mm per week three times in this model. In this study, a group of 14 days was made (early into the chronic phase) and the 21 day group had a chronic process for 1 week, that period can be categorized as a chronic process and CSM is a chronic process. By examining motor scores, histological examination and immunohistochemistry of the spinal cord, this model efficiently produces myelopathy. The distribution of microglia expressing GFAP, S100-β, and Neurofilaments were observed by immunohistochemical techniques.

**Results:**

There was a significant difference in the number of cells expressing GFAP between the control group and the 21-day compression group (p = 0.001). There is a decrease in S100-β expression of spinal cord tissue after receiving compression exposure. There was a significant difference in the number of cells expressing NF between the control group, the 14-day compression group (p = 0.04) and 21-day compression group (p = 0.04).

**Discussion:**

Neurons have the intrinsic ability to regenerate after injury, although not spontaneously. Cervical spondylotic myelopathy causes permanent neurological disorders, partly due to glial scar formation consisting of astrocytes and microglia. The difference between our study and previous research methods is that we perform compression of the spinal cord in stages (0.5 mm, 1.0 mm & 1.5 mm) so that it is more like the natural occurrence of chronic spinal cord compression.

**Conclusion:**

An increasing of GFAP value in this study indicates the presence of astrocyte activity which can be associated with chronic spinal cord injury. There is a decrease in S100-β expression of spinal cord tissue neuron cells after receiving compression exposure. The expression of NF decreased indicating degenerative axons.

## Highlights

• Cervical spondylotic myelopathy in rabbits model.
• Cervical spondylotic myelopathy primary psychological injuries caused by static and dynamic forces.
• The expression of GFAP, S100-β and NF-H in the CSM model.

## 1 Introduction

Cervical spondylotic myelopathy (CSM) is a form of nontrauma-induced spinal cord injury in adults estimated at 54% [1,2]. Cervical spondylosis is the most common cause of myelopathy in the cervical due to chronic compression of the spinal cord in patients aged 55 years or older, only about 10% of the total cases of cervical spondylosis progress to myelopathy [3]. A prospective study found CSM to be the most frequent diagnosis (23.6%) of 585 patients visiting UK Hospital with paraparesis or tetraparesis [4]. The most significant biological processes in the development of CSM are ischemia, blood-spinal cord barrier (BSCB) disorders, chronic inflammation of the neurons and apoptosis. In experimental animals that are given chronic compression on the spinal cord causes pathological and molecular biological changes to CSM [5].

The pathophysiology between spinal cord injury and cervical myelopathy has been known in parallel entity. It has been proposed that primary psychological injuries caused by static and dynamic forces including compressed, pinched, and pulled out inducing secondary injuries at the molecular level [6]. The pathology of spondylotic myelopathy remains unclear because there is no suitable experimental animal model for the study. In this study, we examined the rabbit model of spondylotic myelopathy, in which the disk and facet maintained the cervical spine mobility, and compression with a laminar screw was given 0,5 mm per week three times. In this animal model, a repetitive minor injury causing myelopathy resulted in movement weakness. In the repetitive minor injury model, it is allowed to occur because after the compression screw the rabbit is free to move without a cervical collar (according to the theory of dynamic factors causing myelopathy). By examining motor scores, histological examination and immunohistochemistry of the spinal cord, we can confirm that this model efficiently produces myelopathy.

## 2 Methods

### 2.1 Experimental animal

New Zealand white rabbits 12 weeks of age, weight: 2.6–3.0 kg (average: 2.9 kg), males were used in this study. Animals are given diet and water in the conventional laboratory. The room temperature is around 16–20 °C with a light-dark cycle of 12 h. This study has obtained permission from the ethical committee of the Medical Faculty of Universitas Sumatera Utara, Medan, Indonesia.

### 2.2 Experimental group

This study used 15 rabbits divided into three groups. The first group (n = 5) was a control group, performed a skin incision, paraspinal muscle dissection and lamina hole drill and no laminar screw was installed. The second group (n = 5) performed compression of the spinal cord with a laminar screw, terminated on day 14. The third group (n = 5) performed compression of the spinal cord with a laminar screw terminated on day 21.

In this study, a group of 14 days was made (early into the chronic phase) and the 21 day group had a chronic process for 1 week, that period can be categorized as a chronic process and CSM is a chronic process.

### 2.3 Surgical procedures

The rabbit was anesthetized using 50 mg/kg of Ketamine hydrochloride (Pfizer) and 10 mg/kg of Xylazine (Bayer) [7]. Rabbit in prone position, shaved in the posterior cervical area, disinfect with 10% betadine, sterilized with cloth cover, C4–C6 midline posterior cervical skin incision, small retractors was used, palpation of spinous processes, C5 paraspinal muscle dissection, identification of lamina. One hole is made in the lamina C5 midline position using a high speed diamond drill bur (3 mm in diameter), until it penetrates the lamina (2 mm thick lamina), the burr hole is tappered at 4 mm, then the lamina hole is inserted into a screw (stainless steel) with a diameter of 4 mm and a length of 10 mm, until the entire thickness of the lamina, on the 1st day the compression is given 0.5 mm (by turning the screw 180°), on the 7th day, the screw is turned 180° again (total compression is 1 mm), on the 14th day the screw is rotated another 180° (the total compression is 1.5 mm), after the installation of screw the skin was sutured. The position of the screw head is 0.5 cm below the skin, easily felt, so that in the 2nd and 3rd procedures, it is enough to open one skin suture and the screw is turned, the repeated procedure is carried out by sterilization and the same anesthetics method [[8], [9], [10]].

### 2.4 Motor function evaluation

Motor function was evaluated by using the modification of Tarlov's classification [7] (Table 1). Evaluation was made before and immediately after the surgery, and once a week thereafter.

**Table 1:** Tarlov's classification.

Grade	Motor characteristics
0	Unable to have voluntary movements
1	Perceptible movements at join, the hindlimbs follow
2	Good movements at joins, but unable to stand up
3	Can stand up and walk, but unable to start running quickly
4	Normal

### 2.5 Radiology evaluation

Bone CT scan is carried out to confirm the position of the screw in the midline of the lamina C-5, if the screw position is incorrect, the sample is excluded and replaced with another experimental animal.

### 2.6 Tissue preparation and immunochemistry

The tissue of the C5 spinal cord area was taken and fixed with a buffer solution of 10% formalin. After that, dehydration was carried out using graded alcohol (30%, 50%, 70%, 80%, 96% and absolute) for 60 min each. Clearing was used with xylol 2 times for 60 min each. Then the soft paraffin embedding was carried out for 60 min at a temperature of 48 C°. Furthermore, the paraffin is allowed to stand for one day until it becomes a hard block. The next day it was attached to the holder and a 4 μm thick cut was made with a rotary microtome. Followed by the deparaffinization process; The glass object resulted from the paraffin block was immersed in xylol 2 times for 5 min each. After that, rehydration using serial alcohol (absolute, 96%, 80%, 70%, 50% and 30%) for 5 min each. Then rinsed in H2O for 5 min. Then the process of staining the slide was washed with PBS pH 7.4 for 5 min. Then stained with Hematoxillin for 10 min. After that, soak it in tap water for 10 min. Then rinsed with dH2O. Dehydrated with alcohol 30% and 50% respectively for 5 min. Then stained with Eosin solution for 3 min. Then rinsed with 30% alcohol. Washed with H2O for 5 min and then dried. Then do the mounting with a stick and cover with a glass cover.

### 2.7 Immunochemistry protocol

The distribution of microglia expressing GFAP, S100-β and NF was observed by immunohistochemical techniques. Paraffin block containing spinal tissue was cut to a thickness of 4 μm using a microtome, then deparaffinized with xylol. Subsequently, rehydration was carried out with a decreased concentration of ethanol, followed by rinsing with Phosphate Buffer Saline (PBS) for 3 × 5 minutes. The tissue preparations were then incubated in DAKO® Buffer Antigen Retrieval in a microwave at a temperature of 94C for 20 min and followed by cooling at room temperature for 20 min. The next step, the preparation was washed with PBS for 3 × 5 minutes, and incubated in a peroxidase block (Novocastra®) for 20 min. Furthermore, the preparation was washed again with PBS for 3 × 5 min and incubated in Protein Block for 20 min. After that it was washed again with PBS for 3 × 5 min and incubated overnight (12–18 h) with primary antibodies: anti GFAP (2E1) paint # sc: 33,763; anti NF (RNF 4020 cat # sc-32729; anti S-100 (S1-61) paint # sc-53438, for 1 h at room temperature, then washed with PBS pH 7.2 for 3 × 5 min and incubated with a solution post primary antibody for 45 min and followed by incubation with Novolink® Horse Radish Peroxidase (HRP) for 60 min at room temperature. After incubation, the preparations were washed with PBS pH 7.2 for 3 × 5 min. Then DAB (diamio benzidine) was applied for 10 min and the preparation was washed with PBS pH 7.2 for 3 × 5 min, then counterstain with hematoxylin (Novocastra). Furthermore, dehydration was carried out using increased concentrations of ethanol. The next process is to do the purification with xylol, then do the mounting.

### 2.8 Immunohistologic evaluation

Calculation of immunohistochemical results using techniques such as those in other study modified for spinal tissue [9,10]. Examination of the number of brown cells in the nucleus or cytoplasm per 20 fields of view in the anterior horn compression area (C5) and cell counts were carried out separately between the two examiners (double blind). Examination and cell counts were performed on each slide in the field of view in the cortex of the spinal cord with 400× and 1000× magnification, for 20 fields of view respectively.

### 2.9 Statistical analysis

Statistical analyzes were performed using SPSS Version 21 for Windows (SPSS Inc., Chicago, IL, USA). To test the significance of differences of the variable expression between the two experimental groups, we performed ANOVA tests. The significance level was defined as p < 0.05.

## 3 Results

### 3.1 Evaluation of animal

Homogeneity test performed using one way ANOVA showed that there was no significant difference in body weight between body weight before treatment and body weight after treatment (p > 0.05). This shows that the animal body weight data has a homogeneous variation. Thus, body weight is not a confounding variable that can affect the dependent variable in this study.

**Fig. 1:**
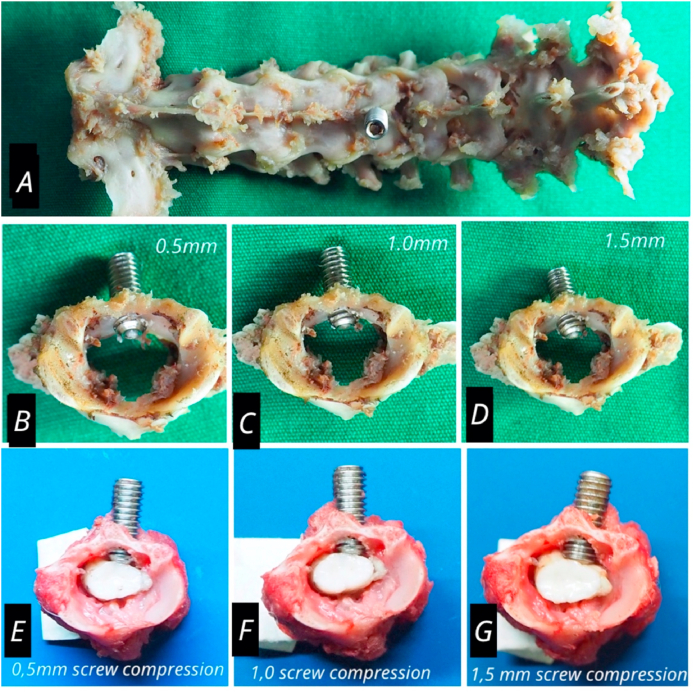
Illustration of rabbit with cervical spine model, rabbit aged 12 weeks, weighted 3 kg, male. (A) Position of laminar screw in midline laminar C5. (B) Compression of the spinal cord on day 1 (C) Compression of the spinal cord on day 7 (D) Compression of the spinal cord on day 14 (E) Position of screw in spinal cord on day 1 (F) Position of screw in spinal cord on day 7 (G) Position of screw in spinal cord on day 14.

Clinical assessment of experimental animals after compression showed no signs and symptoms of acute spinal cord injury. The motor function level of experimental animals given spinal cord compression decreased slowly until day 21. The motor function of experimental animals before being sacrificed in the 14-day compression group was “3″ in the 5 samples and in the 21 day compression group was “2″ at 5 sample. This is different from the experimental animals in the control group for motor function of the five samples “4" (normal) (see Fig. 1).

### 3.2 Evaluation of spinal cord specimens

**Fig. 2:**
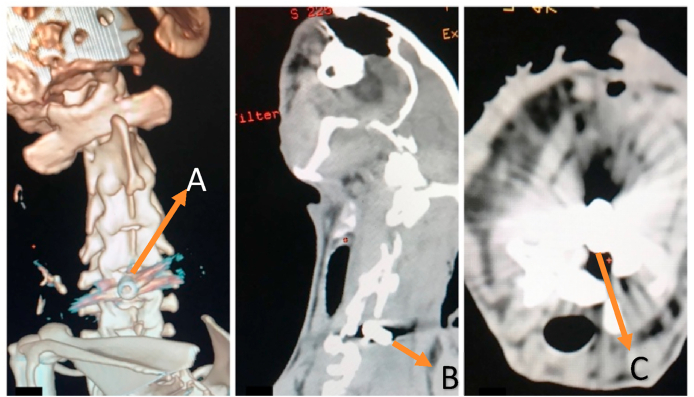
(A) 3D cervical spine shows screw in midline lamina C5 (B) CT Scan Cervical Spine Lateral view shows screw in spinal canal. (C) CT Scan cervical axial view shows screw in midline lamina C5, tip of screw in spinal canal.

**Fig. 3:**
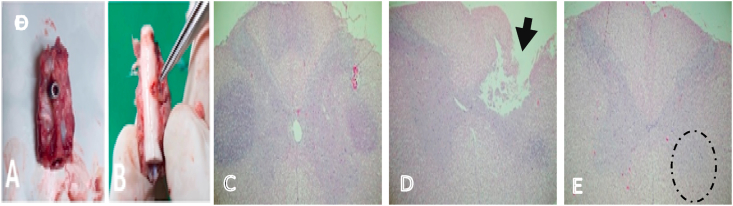
Results of organ isolation and histochemical staining of spinal tissue (A) C4/5/6 cervical vertebra, (B) Spinal cord and screw compression area, (C) Axial cut of spinal cord area (C5) in control group, (D) Spinal cord compression area (C5), (E) The area examined is the anterior horn.

In the area of compression, the spinal cord was seen flattened in the anterior-posterior direction indicating chronic compression (Fig. 3), histological evaluation by hematoxyllin-Eosin staining showed lesions characteristic of chronic myelopathy, neuronal ischemic change, changes in anterior horn cells, edema and ischemic (see Fig. 2).

### 3.3 Glial fibrillary acidic protein (GFAP)

**Fig. 4:**
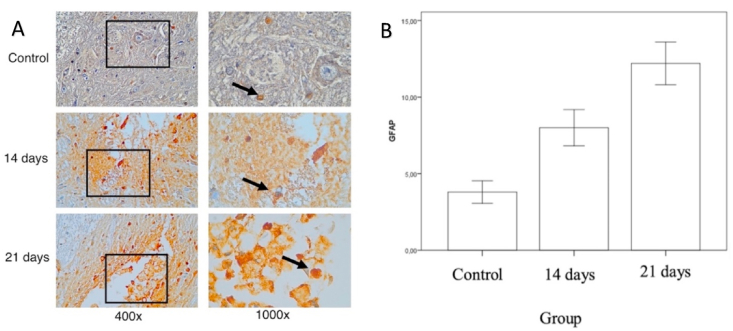
(A)Immunohistochemistry of the number of microglia cells expressing GFAP of the spinal cord tissue, marked in brown on the cell membrane (black arrow) (B) Mean by Standard Deviation of the number of microglia cells expressing GFAP in the control group, the compression group 14 days and 21 days. (For interpretation of the references to colour in this figure legend, the reader is referred to the Web version of this article.)

Neurons have the intrinsic ability to regenerate after injury, although not spontaneously. Cervical spondylotic myelopathy (CSM) causes permanent neurological disorders, partly due to glial scar formation consisting of astrocytes and microglia. This study examined the role of glial injury to the spinal cord due to compression, it appears that chronic compression of the spinal cord causes an increased GFAP expression in the spinal cord tissue, especially in the compression area. The average number of cells that expressed GFAP (Fig. 4) in the control group was 3.8 ± 1,64, the compression group 14 days was 8.00 ± 2.64, the compression group was 21 days 12.20 ± 3,11. There was no significant difference in the number of cells expressing GFAP between the control group and the 14-day compression group (p = 0.06), there was a significant difference in the number of cells expressing GFAP between the control group and the 21-day compression group (p = 0.001). There was no significant difference in number of cells expressing GFAP expression between the compressed group for 14 days and the compressed group for 21 days (p = 0.06). There is a significant difference in number of cells expressing GFAP expression between the control group and the compressed group for 21 days.

### 3.4 S100-β

Astroglial calcium-binding protein (S100-β), a tissue-specific expression of calcium-binding protein [11], has been reported to be significantly increased in serum or cerebrospinal fluid after experimental or clinical spinal cord injury (SCI) [12,13]. This study examined the expression of S100-β by immunoperoxidase technique which was performed by visulalization using DAB. It appears that the expression of S100-β is seen in motor neuron cells in the anterior horn area. By using immunohistochemical results calculation techniques modified for spinal tissue, this study has completed counting the number of neuron cells in the spinal cord tissue, which express S100-β [9,10].

**Fig. 5:**
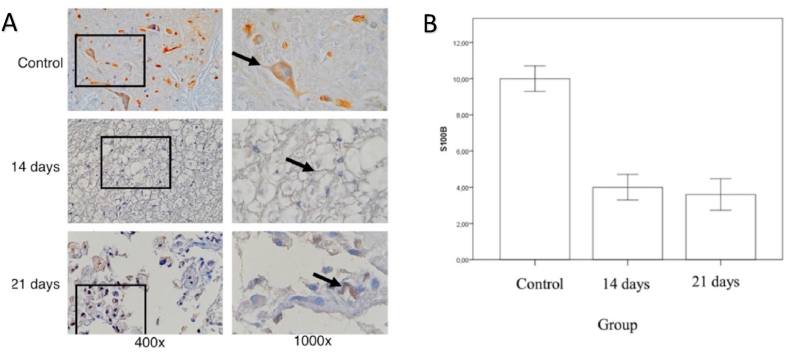
(A) Immunohistochemistry of the number of neurons in the spinal cord tissue expressing S100-β, marked in brown in the cell cytoplasm (black arrow). (B) Mean by Standard Deviation number of spinal neuron cells expressing S100-β on control group, compression of 14 days and 21 days. (For interpretation of the references to colour in this figure legend, the reader is referred to the Web version of this article.)

The mean number of cells expressing S100-β (Fig. 5), shows that in the control group the mean S100-β was 10 ± 1.58, the compression 14 days group was 4.00 ± 1.58 and the compression 21days group was 3,6 ± 1.94. Using a mean difference analysis SPSS 21. It appears that there is a decrease in S100-β expression of spinal cord tissue neuron cells after receiving compression exposure. A significant reduction was seen in the compression exposure group with observations after 14 days compared with the control, as well as the compression exposure with observations after 21 days compared with the controls. Meanwhile, between exposure groups 14 and 21, the expression of S100-β did not appear to show a significant difference.

### 3.5 Neurofilaments (NF)

Proper evaluation of the severity of spinal cord injury is important for developing new therapies. Although several biomarkers in cerebrospinal fluid have been tested, several analyzation of blood samples have been reported. A new biomarker for axonal injury, the phosphorylated form of the high molecular weight neurofilament subunit NF–H (pNF-H), has been reported to increase in blood from the rodent SCI model [14]. The aim of this study was to localize the pNF-H spinal cord tissue modeled with chronic injury.

**Fig. 6:**
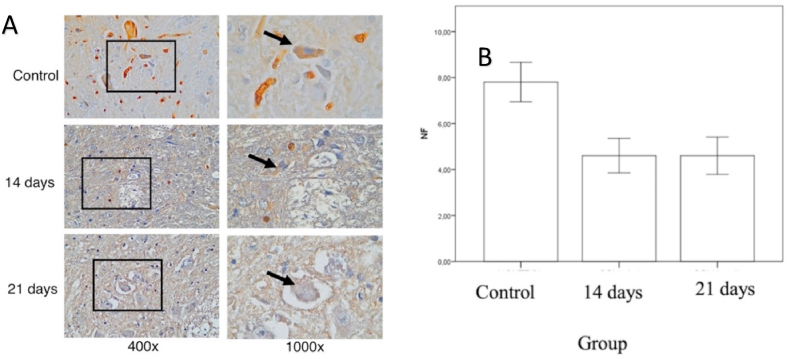
(A) Immunohistochemistry of the number of neurons in the spinal cord tissue expressing NF–H, marked in brown in the cell cytoplasm (black arrow). (B) Mean by Standard Deviation of the number spinal neurons expressing NF–H in the control group, compression of 14 days and 21 days. (For interpretation of the references to colour in this figure legend, the reader is referred to the Web version of this article.)

The average number of cells that expressed NF (Fig. 6) in the control group was 7.8 ± 1.92, the compression group for 14 days was 4.60 ± 1.67 in the compression group for 21 days, 4.6 ± 1.81. There was a significant difference in the number of cells expressing NF between the control group and the 14-day compression group (p = 0.04), there was a significant difference in the number of cells expressing NF between the control group and the 21-day compression group (p = 0.04). There is no significant difference in the number of cells expressing NF between the 14-day compression group and the 21-day compression group (p = 1.00).

## 4 Discussion

Mechanical compression is the corner stone of spinal cord dysfunction in CSM. Mechanical compression can also lead to ischemia and hypoxia, which would result in spinal cord dysfunction, similar to that found in acute traumatic spinal cord injuries. The compression can be caused by static and/or dynamic factors. The static factors refer to structural spondylotic abnormalities such as disc degeneration, which result in cervical canal stenosis. The dynamic factors include changes to the normal cervical spine biomechanics and tensile stresses transmitted to the spinal cord from the dentate ligaments, which attach the lateral pia to the lateral dura [15,16].

The increased association of CSM with aging raises the issue whether anchoring of the cervical spinal cord by dentate ligaments provides tensile friction to cause microtrauma of the spinal cord, or whether the changing stiffness of the neural tissue and extracellular matrix (ECM) in the spinal cord can possibly make the spinal cord stiffer and susceptible to repetitive micro-injury with progressive age [17].

Cervical spondylotic myelopathy is a complex process, but it is a new area of research that is developing restorative neurological methods and is increasingly promising. Treatment of spinal cord injury not only treats neurological injury, but also all secondary complications in other organ systems whose regulation is impaired after CSM. To some extent, CSM rehabilitation is focused on returning to community and functional goals, that is - most importantly - regardless of whether those goals can be achieved through some compensatory mechanism or because of an evolving effort to induce plasticity and neurological recovery. In order to expand CSM rehabilitation study, researchers tried to study the development of a CSM model in rabbits, by observing the expression of GFAP, S100-β and NF–H in the CSM model network compared to non CSM tissue.

In a study presented by Hol and colleagues states that astrocyte reactive (astrogliosis) causes overexpression of GFAP which can be found in various CNS diseases such as spinal cord injury, stroke and neurodegenerative diseases such as spondylotic myelopathy [14]. In a mouse study by Vijayan, VK et al. [18], in spinal cord injury, it was found that increased GFAP expression can be found on the first to 30 days of compression of the spinal cord and correlated with hypertrophy and astrocyte cell proliferation, this is considered a reliable indicator of the process of damage to spinal astrocytes cord resulted in CSM. In this study, using the immunoperoxidase technique, it appears that the development of a CSM model in a rabbit model with laminar screw compression, it appears that up to the 21 day there is an increase in GFAP expression in the astrocyte cells of the spinal cord.

In line with studies, an increase in GFAP indicates the presence of astrocyte activity or progression which can be associated with chronic spinal cord injury. Research found that astrocytes also function to maintain the balance of BBB (blood-brain barrier)/BSCB (blood spinal cord barrier) and participate in various immune system reactions so that GFAP expression is considered a gliotic process due to inflammation or injury and GFAP can be markers of trauma and degenerative disease of the CNS [19]. Astrogliosis of the spinal cord causes scar formation, a barrier that blocks axon regeneration and causes myelopathy as shown in Fig. 3 in this study. To support these results, this study also examines the expression of S100-β in the same area. S100-β is a calcium-binding protein found in glial cells and has previously been established as a marker of brain injury, S100-β has a wide variety of homeostatic activities including regulation of calcium flow, cell proliferation and differentiation, enzymatic/metabolic activity, and MAP stabilization. S100-β is a structural marker that has been reported to potentially predict CSM recovery in various studies.

Research conducted by Low et al. using ELISA revealed increased serum S100-β levels 6 h after injury in 30 mice that underwent CSM when compared to a control group receiving laminectomy alone. However, 24 h after injury there was no significant difference in S100-β concentration between sham and injured mice. In another study conducted, the expression of S100-β reached a peak 24 h after spinal cord injury and thereafter continued to decline. Animal studies found S100-β expression peaks at 24–72 h as a manifestation of acute injury. With the CSM model, which the researchers developed - it appears that - the expression of S100-β decreased significantly after CSM treatment in a rabbit model of spinal cord compression with a laminar screw. This means that the expression of S100-β cannot serve as a marker of CSM in the chronic phase. In order to support the hypotheses that have been built, in relation to the CSM model, this study also analyzes the presence of NF. Neurofilament (NF) is a cytoskeletal protein that is abundantly and uniquely expressed in the cytoplasm of axonal fibers in the CNS. NF regulates signaling and transport systems on axons and has become a focus in neurological disorders due to extracellular accumulation of NF. There are three types of NF: Neurofilament-light (NF-L), medium (NF-M), and heavy (NF–H) chain [20,21].

NF–H will be released from the cytoplasm of damaged neurons in traumatic spinal cord injury. In addition, in the development of secondary injury, when apoptosis and inflammation of the nerves peak, NF is thought to escape extracellularly along with other cytoplasmic components. Therefore, NF loss is hypothesized to potentially indicate the severity of nerve cell loss in SCI as well as the degree of damage at the secondary stage. In this study, it was found that the NF expression decreased on the 14th day of spinal cord compression and continued to decrease on the 21st day, this result is in accordance with a study conducted in rabbits, they found only 6.6% CSM model specimens expressing NF while non CSM (control) 93.3%. Another study in humans with spinal cord injury found that NF expression at 12 h after complete injury continued to decrease and was still detectable until day 21, according to this study it was still found until 21 day compression of spinal cord [20,21]. In previous studies we found significantly lower NF expression in myelopathy compared to normal spinal cord. The stability of neurons, cytoskeleton and its components (actine filaments, neurofilaments and microtubules) is maintained by NF. Neurofilaments are important structures of white matter and represent a very important group of protein structures that support the axon architecture of the central nervous system and peripheral nerves. In this study we found that the expression of NF decreased as an indication of degenerative axons, this is also often found in human CSM.

## 5 Conclusion

CSM is a chronic condition in which there is repetitive minor injury to the spinal cord (dynamic injury) and also slow progressive chronic compression (static injury). In this study we are performing compression to animal model causing CSM condition and then we measure the GFAP, S–100B β and Neurofilament Value.

An increase in GFAP indicates the presence of astrocyte activity or progression which can be associated with chronic spinal cord injury. In this study, it appears that the development of a CSM in a rabbit model with laminar screw compression, there is an increase in GFAP on 21 days group.

Astroglial calcium-binding protein (S100-β), a tissue-specific expression of calcium-binding protein, has been reported to be significantly increased in serum or cerebrospinal fluid after experimental or clinical spinal cord injury (SCI). A significant reduction of S100- β was seen in the compression exposure group with observations after 14 days compared with the control, as well as the compression exposure with observations after 21 days compared with the controls. Meanwhile, between exposure groups 14 and 21, the expression of S100-β did not appear to show a significant difference.

Neurofilament (NF) is a cytoskeletal protein that is abundantly and uniquely expressed in the cytoplasm of axonal fibers in the CNS. NF regulates signaling and transport systems on axons and has become a focus in neurological disorders due to extracellular accumulation of NF. In this study we found that the expression of NF decreased as an indication of degenerative axons, this is also often found in human CSM.

## Ethical approval

Approval has been given by ethical committee of Universitas Sumatera Utara.

## Sources of funding

None.

## Author contribution

Sabri Ibrahim: Author. Wibi RIawan: Co-Author.

## Trial registry number

1. Name of the registry: None
2. Unique Identifying number or registration ID: None
3. Hyperlink to your specific registration (must be publicly accessible and will be checked): None

## Guarantor

Sabri Ibrahim: Author.

Email: sabriibrahimnc@gmail.com.

## Consent

Not Applicable.

## Provenance and peer review

Not commissioned, externally peer reviewed.

## Declaration of competing interest

There is no conflict of interest in this study.
